# The immunoregulation effect of tumor microenvironment in pancreatic ductal adenocarcinoma

**DOI:** 10.3389/fonc.2022.951019

**Published:** 2022-07-28

**Authors:** Jingchang Zhang, Renfeng Li, Shuai Huang

**Affiliations:** The First Affiliated Hospital of Zhengzhou University, Zhengzhou University, Zhengzhou, China

**Keywords:** tumor microenvironment, immunotherapy, immunosuppression, PDAC, immune checkpoint inhibitor

## Abstract

Pancreatic cancer has the seventh highest death rate of all cancers. The absence of any serious symptoms, coupled with a lack of early prognostic and diagnostic markers, makes the disease untreatable in most cases. This leads to a delay in diagnosis and the disease progresses so there is no cure. Only about 20% of cases are diagnosed early. Surgical removal is the preferred treatment for cancer, but chemotherapy is standard for advanced cancer, although patients can eventually develop drug resistance and serious side effects. Chemoresistance is multifactorial because of the interaction among pancreatic cancer cells, cancer stem cells, and the tumor microenvironment (TME). Nevertheless, more pancreatic cancer patients will benefit from precision treatment and targeted drugs. This review focuses on the immune-related components of TME and the interactions between tumor cells and TME during the development and progression of pancreatic cancer, including immunosuppression, tumor dormancy and escape. Finally, we discussed a variety of immune components-oriented immunotargeting drugs in TME from a clinical perspective.

## Introduction

Pancreatic cancer is one of the deadliest malignancies. Despite substantial improvements in the survival rates for other major cancer forms, pancreatic cancer survival rates have remained relatively unchanged since the 1960s. Pancreatic cancer is usually detected at an advanced stage and most treatment regimens are ineffective, contributing to the poor overall prognosis (1). There have been great advances in the diagnosis and treatment of pancreatic cancer in recent years, but clinical data show that only 4% of patients survive five years (2). Pancreatic cancer has been categorized into several types based on their site of origin, difference in pathogenesis, and molecular biology. These are, namely, pancreatic ductal adenocarcinoma (PDAC), pancreatic neuroendocrine neoplasm (PanNEN), acinar cell carcinoma, pancreatoblastoma, and solid pseudo-papillary neoplasm (SPN) (3). PDAC is the most common malignant neoplasm of the pancreas, the conventional type of PDAC which is a tubular adenocarcinoma accounts for 80%-90% of pancreatic cancers, with 60%-70% occurring at the head of the pancreas, mostly in men (4). PDAC is characterized by an immunosuppressive TME accompanied by the major expression of myeloid-derived suppressor cells (MDSCs) and M2 tumor-associated macrophages (TAMs). In contrast, the expression of CD8+ T cells is significantly low. Immunotherapeutic agents target the immunity mediators and empower them to suppress the tumor and effectively treat PDAC. Different targets are studied and exploited to induce an antitumor immune response in PDAC patients (5). The PDAC microenvironment consists of cancer cells, stromal cells, and extracellular components. Stromal cells that contribute to cancer progression are mainly pancreatic stellate cells (PSCs), regulatory T cells (Tregs), MDSCs, cancer-associated fibroblasts (CAFs) and TAMs. These cells and tumor cells can secrete extracellular components, such as extracellular matrix (ECM), matrix metalloproteinase (MMP), growth factors, and transforming growth factor-β (TGF-β), to maintain the TME (6). Recent studies have demonstrated that the TME plays a critical role in PDAC progression (7). Tumor immunotherapy is a therapeutic method to control and eliminate tumors by restarting and maintaining the tumor-immune cycle and restoring the body’s normal anti-tumor immune response, including monoclonal antibody immune checkpoint inhibitors, therapeutic antibodies, cancer vaccines, cell therapy and small molecule inhibitors, etc. In recent years, the good news of tumor immunotherapy has been continuously, and it has demonstrated strong antitumor activity in the treatment of pancreatic cancer (8–10). Tumor immunotherapies can enhance the immune system of the body, increase the specific recognition and memory of tumor cells, reduce the toxic and side effects on the body, and then achieve durable cure (10). Immunotherapy has become a powerful clinical strategy for treating cancer. The number of immunotherapy drug approvals has been increasing, with numerous treatments in clinical and preclinical development (8).

## Immunosuppressive associated cells during the progression of PDAC

### Tregs

**Figure 1:**
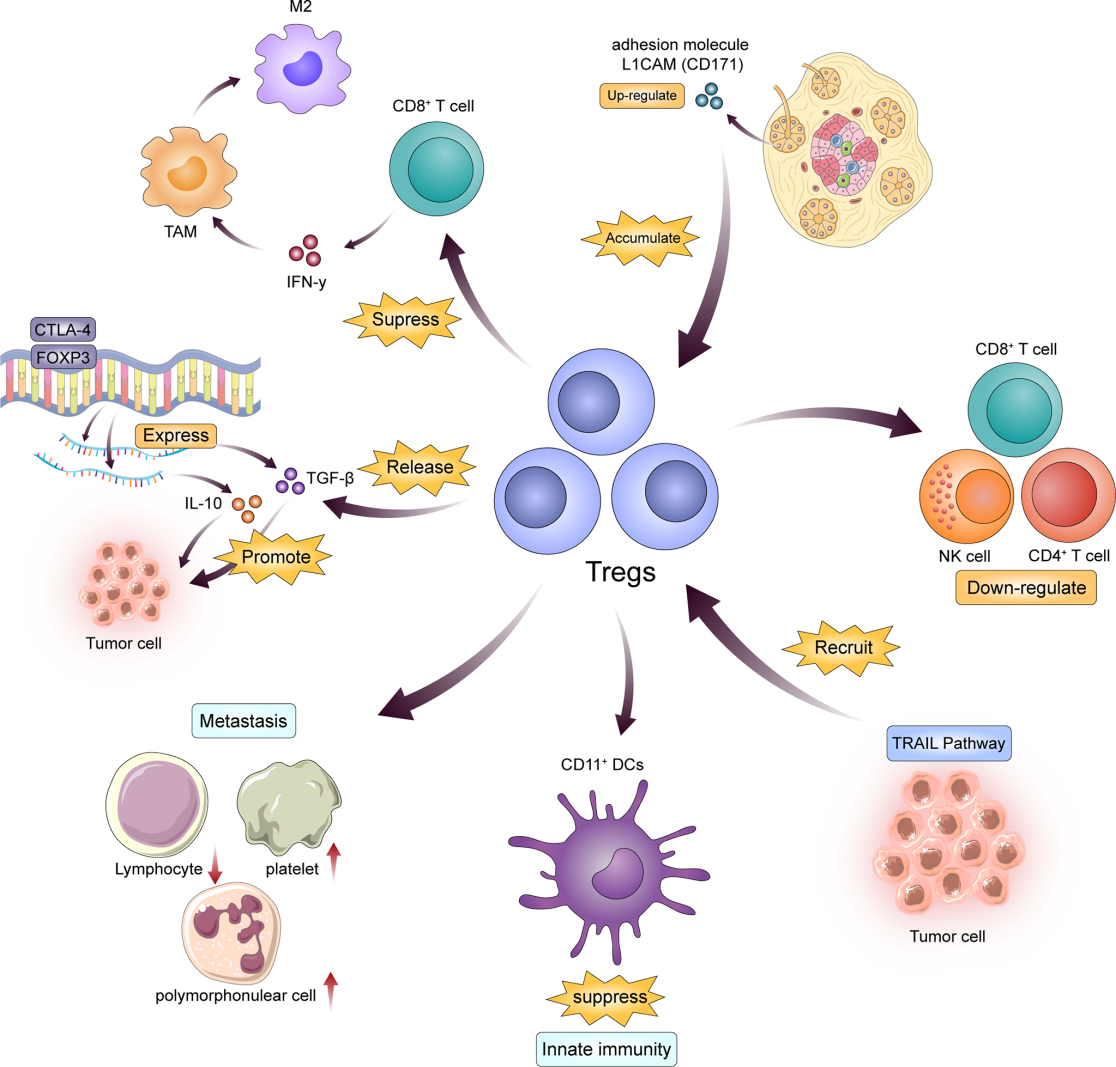
Tregs are the key to immunosuppression in the progression of PDAC. On the one hand, PDAC cells recruit Tregs in a variety of ways; on the other hand, Tregs change the blood environment and maintain the activity of M2-TAMS and DCs by releasing cytokines such as TGF and IL, thus achieving the effect of inhibiting effector T cells.

Tregs, called regulatory T cells, are typically CD4 and CD25 positive, a subtype of T cell. The main function of these cells is to inhibit the proliferation and induction of effector T cells and to maintain autoantigen tolerance (11). Tregs infiltration constitute a prominent feature of PDAC. Tregs is an important part of tumor interstitial immune infiltration, and their presence is associated with poor clinical outcomes in many cancer types. Treg cells are the most potent antitumor immunosuppressants known, inhibiting the activity of CD4+, CD8+ and NK cells. Various mechanisms of Treg cell-mediated immunosuppression include the direct elimination of effector T cells or the acquisition of antigen-presenting cells in competition with effector T cells (12). Tregs release of TGFβ and interleukin-10 (IL-10) have also been suggested as a possible mechanism leading to this type of cellular immunosuppression. However, the exact role of Treg cells in pancreatic tumorigenesis remains largely unknown (13). Jan et al. demonstrated that Treg can confer an immunosuppressive property to their critical target, CD11c+ DC, which suppress immunity against cancer cells. Furthermore, the adhesion molecule L1CAM (CD171) is upregulated in pancreatic duct epithelium during the progression of PDAC and is associated with the accumulation of immunosuppressive T cells in tumor stroma (14). From a clinical perspective, the total number of circulating white blood cells and platelets may have a prognostic impact on patients with PDAC. These patients often have a poor prognosis associated with reduced lymphocyte counts, increased platelet count and polymorphonuclear cell counts (12). To understand the functional role of Tregs in PDAC development, we adopted an *in situ* implantation model. Pancreatic duct epithelial cells (Kras G12D-PDECs) originally representing KrasG12D were injected into the pancreas of C57BL/6 wild-type (WT) mice with the same gene by GFP labeling (GFP-KRAS G12D-PDECS). This model Outlines histologically the preinvasive stage of pancreatic intraepithelial neoplasia (PanIN) that develops and induces a similar intrapancreatic immune response (15). These data indicate that the development of PanIN is accompanied by the progressive accumulation of activated Tregs (14, 15). The expression of Foxp3 and CTLA-4 mRNA in peripheral blood Tregs of patients with advanced and advanced PDAC is higher, and there should be further positive correlation between IL-10 or TGF-β level and PDAC progression (16). Tregs can inhibit the body’s original anti-tumor effects, that is, tumor immunity, by combining multiple cytokines and pathways. Tregs secrete a range of inhibitory cytokines and molecules, including IL-10 and TGF-β, which, as shown in clinical studies, inhibit effector T cell function and result in lysis and inactivation (17). The process of Treg induced effector T cell lysis and inactivation is also involved in granzyme B (18, 19), TRAIL pathway (20) and galectin-1 (21), etc. Studies have shown that PDAC cells can recruit Tregs through the TRAIL pathway and promote tumorigenesis and progression (22). Furthermore, Tregs competitively bind with IL-2 to inhibit proliferation of effector cells involved in antitumor immunity (23). CTLA-4 expressed by Tregs upregulates indoleamine 2, 3-dioxygenase (IDO) pathways in dendritic cells and effector T cells and leads to their dysfunction (24, 25). Tregs also regulated lipid metabolism in M2-like TAMs. Liu et al. found that Tregs inhibited the secretion of interferon-γ (IFN-γ) by CD8+T cells, thereby blocking the activation of fatty acid synthesis mediated by sterol regulatory element binding protein-1 (SREBP1) in M2 TAMs. Thus, Tregs indirectly but selectively maintained m2-like TAM metabolic activity, mitochondrial integrity, and survival. Therefore, there are a positive feedback loop between TAMs and Tregs, which further enhance their immunosuppressive effect in TME (26) (**Figure 1**).

### TAMs

Macrophages derived from monocytes are phagocytes involved in the innate immune system. Due to their plasticity, macrophages are composed of heterogeneous populations of cells with different functional and phenotypic characteristics (27). Based on activation mechanisms, macrophages are classified as M1 (activated by IFN-γ and TLR ligands, expressing higher levels of IL-12, IL-23, MHC II and inducible nitric oxide synthase, with tumoricidal effects) or M2 (activated by IL-4 and IL-13, expressing higher levels of IL-10 and TGF-β, and promote tumor progression) (28, 29). Studies have shown that the M2 phenotype of TAMs have obvious immunosuppressive effect (30). The accumulation of TAMs is associated with the progression of malignant tumor and treatment resistance, especially the immunosuppression of PDAC (31). Overall survival was shorter in patients with high density M2 macrophage infiltration than in patients with low density M2 macrophage infiltration (30). The M2 phenotype of TAMs can contribute to tumor progression by producing multiple mediators that maintain TME. These mediators mainly include growth factors and cytokines which support the proliferation of tumor cells. NF-κ B-mediated protective factors of apoptosis (e.g., IL-1β, IL-6, tumor necrosis factor (TNF)-α, C-C motif chemokine (CCL)2, C-X-C motif chemokine (CXCL)8 and CXCL10) (32, 33); Angiogenic growth factors, such as vascular endothelial growth factor (VEGF), platelet-derived growth factor (PDGF), TGF-β and fibroblast growth factor (FGF) (34–36); And other factors that regulate tissue structure and promote tumor cells migration, invasion and metastasis (37). TAMs lead to PDAC immunotherapy resistance, and ZEB1 is a key cytokine involved in this process in TME. Its main role is to maintain TAMs tumor-promoting function by promoting epithelial-mesenchymal transformation (EMT) of cancer cells. Clinical studies have shown that TAM infiltration in PDAC cells depends on the expression of CCR2 and ZEB1, of which CCL2 and CD74 determine poor prognosis (38). An important process in the therapeutic resistance of PDAC is the interaction between TAMs and cancer stem cells (CSCs), that is, TAMs can be recruited into TME to regulate the start-up state of pancreatic CSCs (39), which secrete IFN-β (40) and other factors to stimulate TAMs to maintain the active state, thus initiating the proliferation and differentiation effect of tumor cells. TAMs can express more Arg1 to interfere with the metabolism of effecting T cells, and TGF-β, IL-10, prostaglandin E2 (PGE2) and other factors released by TAMs can help Tregs recruit and inhibit CD8+T cells (41, 42). TAMs can also induce T cells apoptosis by expressing PD-L1 on its surface, which is similar to PDAC cells and MDSCs (43). TAMs play a dual role as “tumor promoter” and “immune suppressor”, helping to establish a pro-inflammatory microenvironment (44–46). TAMs also disrupts local immune surveillance, Because they indirectly inhibit T cell activity by expressing cell surface proteins or releasing soluble factors that demonstrate immunosuppressive function (e.g., argininase 1 (ARG1), IDO, IL-10, TGF-β) (37, 47) or by recruiting other immunosuppressive cells such as Tregs (48). The interaction between TAMs and immune checkpoint inhibitors and other components of TME leads to the inhibition of immune checkpoints such as PD-1/PD-L1 and CTLA-4, resulting in the elimination of inhibitory signals for T cell activation, thus enabling tumor reactive T cells to overcome regulatory mechanisms and produce effective antitumor responses, known as the expression pattern of checkpoint molecules (49, 50). Phosphatidylinositol 3-kinase γ (PI3Kγ), as a molecular switch, is closely related to the phenotype and classification of TAMs, and its involvement in immunosuppression mainly depends on turning off the “immune-stimulatory program” and turning on the “immunosuppression program” (51). Kaneda et al. showed that PI3Kγ determines the immunosuppressive properties of TAMs. Studies have shown that Tamsin lacks PI3Kγ activity, resulting in the expression of MHC-II and pro-inflammatory cytokines, while reducing immunosuppressive molecules including IL-10 and arginase. This significant change in TAMs also enhanced adaptive immunity of TME and significantly inhibited tumor progression (52). In summary, TAMs interact with a variety of cells in TME, using cytokines and intercellular signal transduction pathways as intermediate bridges to jointly promote PDAC progression and tumor immunity, resulting in treatment resistance and poor prognosis.

### MDSCs

MDSCs represent another group of innate immune cells that suppress both innate and adaptive immunity (13). The marker of MDSCs is CD11b+, CD33+, HLA-DR- in humans. The levels of MDSC and pre-MDSC cytokines in peripheral blood of patients with PDAC are higher, and MDSCs in peripheral blood may be a predictive biomarker of chemotherapy failure in patients with PDAC (53). In addition, cultured bone marrow mesenchymal stem cells have been shown to induce Tregs development, whose function has been discussed previously, and targeted removal of bone marrow mesenchymal stem cell subpopulation GR-MDSC can lead to accumulation of activated CD8+T cells, tumor cell apoptosis, and tumor stromal remodeling (54). In PDAC, MDSCs are recruited to TME by tumor cells, mainly due to the production of granulocyte macrophage colony stimulating factor (GM-CSF) (55, 56). GM-CSF upregulation may be caused by KRAS^G12D^ mutation, which is detected in almost all cases of PDAC. Once inside the TME, MDSCs inhibit effector T cells, affecting several different pathways. For example, they can induce oxidative stress in T cells by producing reactive oxygen species (ROS), resulting in impaired T cell protein translation, leading to a lack of antigen-dependent proliferation (57). In addition, MDSCs inhibit T cell proliferation by depleting L-arginine from TME through a signal transductor and transcriptional activator3 (STAT3) dependent mechanism. Moreover, MDSCs also showed the ability to promote and maintain the development of Tregs to form tumor stroma (54). The process of this response is shown as follows. MDSCs produced by increased mobilization of granulocyte colony-stimulating factor (G-CSF)/IL-3/GM-CSF mediated progenitor cells express cysteine transporter Slc7A11, arginase and IDO are produced to isolate metabolites and ensure the initiation and maintenance of Tregs in tumors, thus directly inhibiting the proliferation of CD8+T cells (56, 58, 59). It can also directly produce carbon monoxide and reactive oxygen species through external stimulation and damage the effector T cells and their receptors to achieve immunosuppression effect (54). In simple terms, in PDAC, tumor cells produce GM-CSF, which promotes the accumulation of MDSCs in TME, thereby limiting T cell response.

### Cancer stem cells

CSCs are subpopulations of small cells that are located between tumor cells and possess stem-like properties, cloning, long-term regeneration and self-renewal (60). CSCs in PDAC have a variety of specific markers, among which the most characteristic are CD44+, CD24+, CD133+ and ESA+ (61–63). It was found that CSCs positive for these specific markers increased tumorigenic potential several times, were more likely to mediate immunosuppression leading to therapeutic resistance and maintained their surface marker phenotypes after repeated passage as xenografts. Clinical data indicate that CD44+ is an important indicator of poor prognosis in PDAC patients (64). Some specific states in TME, such as acidosis and hypoxia, have great influence on the proliferation and differentiation of tumor stem cells, and thus directly affect tumor immunity. And the proliferation of PDAC stem cells and the immunosuppressive effect of PDAC stem cells are mainly dependent on the hypoxia state of TME. Hypoxic regions within tumors provide a favorable ecological niche for cancer cells to acquire and maintain stem cell properties, known as CSCs phenotypes, such as self-renewal, globular formation and metastasis capacity, and an undifferentiated state (60, 65). Pancreatic CSCs are highly heterogeneous in their surface and intracellular markers and in response to chemotherapy, radiotherapy, and hypoxia (66). During tumor progression, the expression of nestin, a pancreatic CSCs marker, is required to drive EMT and promote migration and invasion of PDAC cells. PDAC cells induced nestin expression through TGF-β1/Smad4 pathway under hypoxia. More importantly, elevated nestin expression promotes positive feedback constitutive activation of TGF-β1/Smad signaling by increasing the expression of TGF-β1, TGF-βR1 and TGF-βR2 in nestin-positive PDAC cells (67).

### DCs

DCs from bone marrow are powerful antigen-presenting cells (APCs) with diverse and complex functions (68). After being activated in blood, dendritic cells migrate to lymph nodes, interact with effector T cells, and become the center of immune response, participating in innate and adaptive immunity. In addition, in this process, DCs recognizes endogenous and exogenous proteins associated with PDAC cells and degrades and integrates them, known as antigen internalization, which is presented on the cell surface and binds to native T cells, thereby initiating and regulating adaptive immunity and activating immunity against tumors (69). Traditional DC (cDC) is a branch of DCs lineage, which is closely related to the development of tumors. Studies have shown that some molecules in TME, as well as hypoxia environment and lactic acid inhibit the function of cDC, and specific receptor and ligand binding can target the activation and maturation of DC (70, 71). As mentioned above, the immune-related role of DCs is mainly to sense the microenvironment of tumor cells and form the corresponding adaptive immune response. DCs in PDAC are involved in the immunosuppressive effect of TME by differentiating the differentiation of different CD4+ T helper cells (Th), including Th1, Th2, Th17, through multiple phenotypic and functional heterogeneity subsets (72).

### PSCs

PSCs are stellate stationary stromal cells that appear as myofibroblast-like cells located in the exocrine region of the pancreas (73, 74). PSCs are normally static in the body, and its main role is to participate in normal pancreas secretion, original immune response and maintenance of homeostasis of internal environment, as well as storage of vitamin A (75). Under the stimulation of special environmental factors, such as ROS, growth factors, cytokines, signal transduction factors, etc., PSCs transform into an activated state, which is called the activated state (76). This activation state shift is accompanied by loss of lipid droplets and expression of activation marker α -smooth muscle actin (αSMA) (77). Activated PSCs maintain their activation state through autocrine. Insulin-like growth factor 1 (IGF1), VEGF, PDGF, FGF, and CXCL12 can promote tumor angiogenesis, proliferation, migration, and immunosuppression through paracrine production (78). Studies have shown that activated PSCs can establish an interaction with PDAC cells by secreting cytokines such as TGF-β, IL-6, stromal cell derived factor-1 (SDF-1), hepatocyte growth factor (HGF) and galactose lectin-1, which contribute to promoting the immunosuppressive properties of TME and supporting the aggressibility of PDAC and the main mechanism is to promote EMT of tumor cells, which is mediated by IL-6 (79, 80). PSCs are closely related to the immunosuppression of pancreatic malignant tumors, one of the key points is to block the activation and inhibit the function of lymphocytes. For example, CXCL12 secreted by PSCs significantly reduces the migration of CD8+T cells to the peripheral stroma of pancreatic cancer, resulting in reduced anti-tumor activity of effector T cells (81). PSCs also achieve immunosuppression in PDAC microenvironment through galectin-1 mediated T cell apoptosis and Th2 cytokine secretion (82). Another factor is that PSCs play an immunosuppressive role in TME by combining with other immune-related cells and cytokines. The first pathway is IP-10/CXCL10 (interferon-inducible protein 10/CXC chemokine ligand 10), which is used by PSCs to recruit Tregs (83). In clinical studies, elevated IP-10 levels in patients with PDAC are often associated with poor prognosis and a larger invasive range of pancreatic tumors (84). The second pathway is IL-6/STAT3, through which PSCs induce the differentiation of peripheral blood monocytes into MDSCs, thereby inhibiting the proliferation of effector T cells (85).

### CAFs

In the progression of pancreatic malignant tumors, fibroblasts mainly regulate the invasion of tumor cells, leading to extensive invasion and distant metastasis, but they are also closely related to tumor immunosuppression (86). Clinical studies have shown that PDAC FAP+ fibroblasts have at least two main states: periglandular αSMAhigh myofibroblastic CAFs (myCAFs) and diffusely distributed αSMAlow IL-6–positive inflammatory CAFs (87). Clinical results have shown that myCAFs have a synergistic effect on T cells and inhibit tumor growth, while inflammatory CAFs promote tumor growth and immunosuppressive response by secreting ECM proteins and cytokines such as IL-6, IL-11 and leukemia suppressors (88). The process of immunosuppression is reflected in that these cytokines can activate IL-6R+ malignant cells and myeloid cells, thereby activating STAT3 signaling and promoting tumor growth (89). As mentioned earlier, IL-6 is also strongly associated with cachexia and subsequent poor prognosis in PDAC (90). Targeting IL-6R and IL-11R in mouse models can significantly reduce STAT3 activation and enhance the effect of chemotherapy agents such as gemcitabine in tumor killing effect, suggesting that the interleukin pathway may be a good therapeutic target (91). Ber et al. Confirmed through RNA sequencing analysis that in PDAC, the increased expression of various cytokines produced by CAFs can affect the pathways of ECM remodeling and immune response to varying degrees, supporting their involvement in immunosuppression in PDAC, and ultimately leading to tumor invasion and metastasis (92). Recent studies have shown that Netrin G1 (NetG1) is the promoter of PDAC tumorigenesis in CAFs-related TME, and NetG1+CAFs stimulate immunosuppressive response and promote tumor progression through NetG1-mediated effects on glutamate/glutamine metabolism. In addition, the immunosuppressant effect of NetG1+CAFs are also reflected in the inhibition of natural killer cell-mediated killing of tumor cells, which is controlled by the downstream signaling pathway NetG1, which consists of AKT/4E-BP1, P38/FRA1, vesicular glutamate transporter and glutamine synthase (93). In the microenvironment of PDAC, CAFs can not only participate in the extensive fibrosis of the primary site, resulting in the proliferation of connective tissue, providing conditions for invasion and metastasis, but also participate in the immunosuppressive reaction by acting on a variety of immune cells such as effector T cells and NK cells, exacerbating progression.

### Bregs

At the invasion stage of PDAC, a variety of immune cells participate in the tumor immune response of the body. Like Tregs, B cells also generate regulatory B cells (Bregs) through their own activation and release chemokine CXCL13 to infiltrate the microenvironment (94), which is both tumor-promoting and tumor-suppressive. One risk factor for PDAC is long-term chronic pancreatitis (95). An important microenvironmental change in chronic pancreatitis is extensive B lymphocyte infiltration. In human PDAC samples, PDAC patients with higher B cell content in TME have significantly shorter survival (96). When constructing mouse models, we found that during this process, extensive infiltration of Bregs increased the expression of IL-1β and decreased the activity of CD8+ T cells, promoting tumorigenesis (97), while reverse expression of PD-L1 and IL-35 in Bregs supported immune escape of tumor cells (98, 99). In addition, one of the characteristics of the occurrence and progression of PDAC is matrix reaction. In the study, it was found that Tregs produce PDGF-B, a pro-fibrosis molecule, and stimulate collagen generation through fibroblasts, resulting in massive production of CAFs to maintain the activation of tumor matrix and promote tumor metastasis (100). Bregs also secretes immunomodulatory cytokines such as IL-35 and IL-10, which induce Tregs production, stimulate tumor proliferation and promote local angiogenesis (101). Therefore, understanding the risk factors underlying inflammation in PDAC will have a profound impact on subsequent treatment regimen and prognostic monitoring.

## Immune-related cellular biology in the progression of PDAC

### Pre-metastatic niche

PDAC cells can facilitate the colonization of tumor cells by forming a supportive microenvironment at the site of metastasis through a series of cells and cytokines such as circulating tumor cells (CTCs), which is called the premetastatic niche. The formation and stable state of pre-metastatic niche is the key to the progression of PDAC and can also induce tumor dormancy at the metastatic site, and thus relapse at the metastatic site. Intercellular communication is the key to niche formation before metastases, the most common of which is liver metastases (102). The formation of an active environment is the basic mechanism that ensures the successful arrival and realization of tumor cells before they reach the secondary distal site. The primary tumor interacts with the environment of the metastatic organ to create an abundant microenvironment, while the spread of tumor cells requires many molecular and cellular changes in TME, thus providing fertile soil for the formation of secondary tumors in distant organs. The primary tumor secretes some essential soluble molecules such as TDSFs including tumor necrosis factor alpha (TNF-α, TGF-β, and VEGF along with extracellular vesicles (EVs) that are required for the preparation of distant receptive sites for pre-metastatic niche formation and organ-specific metastasis or organotropism (103). CAFs and tumor exosomes play important roles in regulating ECM remodeling for the preparation of the pre-metastatic niche. CAFs which are induced by hypoxic condition produce TGF-β2 that can preserve the stemness of the niche (104, 105). By secreting IL11, CAFs support distant metastasis through activating Glycoprotein 130 (GP130)/STAT3 signaling in PDAC cells (106). Also, increased STAT3 activity in CD11b+myeloid cells, motivates the local invasion of primary tumor cells into the blood or lymphatic vessels to move towards the metastatic sites and create pre-metastatic niche (107). Studies have shown that exosomes derived from PDAC can induce the formation of pre-metastatic niche in liver. The mechanism of pre-metastatic niche formation is that the uptake of PDAC exosomes by Kupffer cells leads to the secretion of TGF-β and up-regulation of fibronectin production by hepatic stellate cells, thus enhancing the recruitment of bone-derived macrophages. During this process, macrophage migration inhibitory factor (MIF) plays an important role. Studies have found that blocking MIF highly expressed in PDAC can effectively prevent the formation and metastasis of liver pre-metastatic niche (108). In the microenvironment of PDAC, a variety of immune-related cells are involved in the formation of liver pre-metastatic niche. TAMs secrete granular proteins to activate stellate cells to differentiate into myofibroblasts, forming a hepatic fibrosis microenvironment and supporting the growth of metastatic PDAC (109). CXCR2 is involved in the formation of pre-metastatic niche in the liver of PDAC through G-protein-coupled receptors that control neutrophil and MDSCs migration (110). The property of liver metastasis in PDAC is mainly reflected in hematopoietic stem cell (HSC) migration and homing, which is initiated by cancer-initiating cells (CIC) (111). CXCR4 and CXCL12 are key to tumor cell migration and directly affect the formation of pre-metastatic niche. Increased CXCL12 expression in hypoxic tissues is important for the homing of tumor stem cells in circulating tissues (112), CAFs secrete CXCL12, attract CXCR4-expressing tumor cells and endothelial progenitor cells, thereby promoting angiogenesis in the liver’s pre-metastatic niche (113, 114). Another important procedure is through the receptor tyrosine kinase Met (115), which shows that HGF and Met can drive the mobilization and migration of tumor stem cells in tissues (116, 117). CD44+CIC contributes to the activation of the Met signaling cascade (118), HGF-Met axis participates in angiogenesis through HGF expressing mesenchymal stem cells, and c-Met expression at high levels in CIC promotes liver implantation and metastasis of PDAC (119). In TME, pre-metastatic niche formation relies on EVs for extensive communication between tumor cells and others. The EV-capsuled factors not only enter circulation and reach distant organs to construct a pre-metastatic niche, but also facilitate tumor growth locally, include regulating angiogenesis, cellular metabolism, metastasis, cell survival, immune regulation and therapeutic resistance (120). At the level of gene expression, Rab27a GTPase is overexpressed in advanced tumors during the establishment of pre-metastatic niche and is used to regulate vesicle transport, which is related to the non-cellular autonomous control of tumor growth and metastasis. In addition, down-regulation of Rab27a can increase the expression of genes related to the EMT pathway and alter the characteristics of autonomous invasion of tumor cells. And these reveal that Rab27a can play divergent roles in regulating pro-metastatic propensity of PDAC cells: by generating pre-metastatic environment at the distant organ sites, and by suppressing invasive properties of the tumor cells (121). However, the specific mechanism of pre-metastatic niche formation remains unclear. Just as CTCs is the “seed” and the organ that is about to undergo distant metastasis is the “soil”, CTCs change the tissue microenvironment by secreting a variety of cytokines before planting to create conditions for tumor cells growth, such as hypoxia and acidosis (122).

### Tumor dormancy

When PDAC cells spread to distant organs such as liver and lung, due to the limitation of growth microenvironment, tumor cells will be in non-proliferation or equilibrium state, that is, cell proliferation rate is equal to its death rate, which is called tumor dormancy. The mechanisms of PDAC cells dormancy can be divided into three categories: cell dormancy, angiogenic dormancy, and immune-mediated dormancy. The main processes include the cellular mechanisms that push a small number of DTCs (disseminated tumor cells) into a dormant state, the balance between tumor angiogenesis related cell proliferation and death, and the immune system maintaining a constant number of proliferating tumor cells (123–125). In addition, PDAC cells can sense changes in microenvironment and determine changes in dormancy state through expression of intracellular homologous ligands and receptors, thus promoting or inhibiting the growth of PDAC cells at distant metastasis sites (126). Studies have shown that the main significance of tumor dormancy is that it was demonstrated that they are a cellular reservoir for a swift relapse of pancreatic cancer following oncogene reactivation (127). DTCs and CTCs are the main mechanisms for tumor dormancy. The first two types of tumor cells also participate in the occurrence of immune escape. Among them, normal stem cell quiescence, extracellular and stromal microenvironment, autophagy, and epigenetics are the mechanisms that determine tumor cells dormancy (123). Clinical studies have found that PDAC cells in distant metastatic sites often remain clinically asymptomatic due to the dormancy of tumor cells, resulting in occult onset and ineffectiveness of conventional treatment (128–130). Under the pressure of high energy consumption of tumor cells and insufficient nutrition supply of local tissues, cancer cells secrete factors that inhibit the PI3K pathway, and this nutrient deficiency leads to the loss of tumor adhesion, further promoting short-term growth stagnation, and ultimately leading to stasis and autophagy induction. Therefore, reduced PI3K–AKT signaling has been linked to dormancy-like phenotypes (131–133). Related studies suggest that autophagy may be a survival mechanism during dormancy. Autophagy of tumor cells inhibits PI3K-Akt signaling while maintaining dormancy DTCs metabolic adaptability. The autophagy signaling mechanism can integrate stationery and survival signals to promote damage repair so that PDAC tumor cells can survive and develop therapeutic resistance during conventional chemotherapy as well as immunotherapy and targeted therapy (134–136). Although reduced mitogenic signaling can trigger quiescence, specific kinases such as dual specificity tyrosine-phosphorylation-regulated kinase 1B (DYRK1B) can actively induce this state (137). DYRK1B blocks the G0/G1/S conversion mechanism proteins, including cyclin D1, CDK4, and P27, in pancreatic malignant tumor cells (138, 139). DYRK1B also coordinates survival through antioxidant responses, inhibits and specifically kills resting pancreatic cancer cells through specific pathways, but does not kill normal resting cells (140). In conclusion, DYRK1A and DYRK1B may be markers of PDAC dormant cells. In addition, Lin et al. demonstrated that Kras mutation combined with c-MYC overexpression in PDAC tumor cells is closely associated with tumor dormancy in distant metastatic lesions (127). The dormancy of PDAC tumor cells does not mean the stagnation of cell cycle, but the existence of a dynamic equilibrium. Under the influence of TME and stem cells and corresponding cytokines, the cells were randomly transformed into activated states. Studies have shown that signals in normal stem cell niches seem to regulate dormancy, it also plays an important role in removing tumor dormancy. However, in dormant DTCs cell cycle arrest is coupled to a persistent but latent form of tumor-initiating or pluripotent capacity that provides an adaptive and survival advantage that eventually fuels tumor growth (114, 141). Tumor dormancy is closely related to immune cells and is involved in the immunosuppressive effect. T cell-induced tumor dormancy also involves crosstalk with endothelial cells and angiogenesis inhibition. Studies have shown that CD4+ T cell mediated antitumor effect is closely related to inducing tumor dormancy, and tumor progression is inhibited by its independent generation of tumor necrosis factor receptor 1 (TNFR1) and interferon -γ (IFN-γ) signals (142). IFN-γ signaling is a powerful inducer of growth stagnation and dormancy, acting mainly by removing immunosuppression of effector T cells. Meanwhile, IFN-γ can irreversibly induce tumor cell senescence and transform into a significant growth arrest phenotype in the T-antigen-induced PDAC model (143). Understanding tumor dormancy is important because dormant cells may be the source of tumor recurrence. Studies have shown that, overexpression of the Notch signaling ligand DLL4 (delta-like 4) in endothelial cells can promote T-ALL (T-cell acute lymphoblastic leukemia) cells to exit dormancy by binding to the Notch3 receptor on T-ALL cells, and this overexpression of DLL4 can be induced by VEGF (144). Regulating endothelial cell and dormancy DTCs interactions with VEGF inhibitors may be a new approach to prevent dormancy withdrawal. In addition to endothelial cells, other cell types may also facilitate the transition from dormancy, for example, recruitment of blood vessels through tissue factor signaling (CD105+) and myeloid cells (CD11b+ and F4/80+) can terminate the dormancy of tumor cells (145, 146). In this theory, inhibition of tumor cell activation in the dormant state can effectively improve the later survival rate of PDAC patients (126).

## Signaling pathways important for PDAC immunosuppression

**Figure 2:**
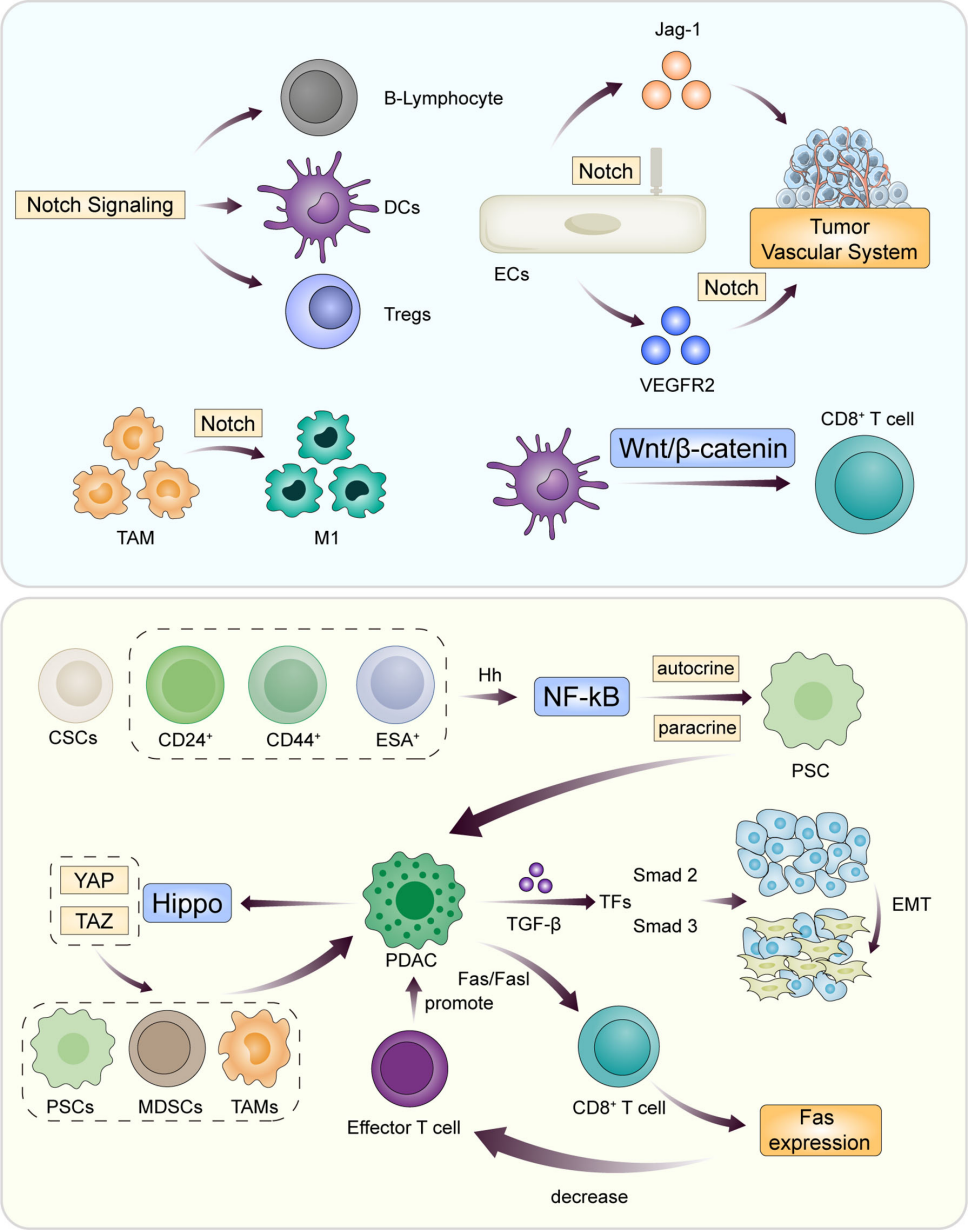
During the progression of PDAC, the innate immune system of the body is activated and antagonizes the immunosuppressive mechanism of tumor cells. Intercellular information transmission involves multiple complex signaling pathways through paracrine and autocrine. Classic signaling pathways include Notch, TGF-β, Wnt, Hippo, Fas/FasL, and Hh. The core of the pathway is to promote or inhibit the function of B cells and effector T cells by changing the cellular components of TME.

Immunosuppressive effect in microenvironment is an important factor in the progression of PDAC. In the process of immunosuppression, a variety of cells involved in immune function transmit information through cytokine pathways or intercellular transduction pathways, such as Notch, TGF-β, Wnt, Hippo, Fas/FasL, Hh, et. These signaling pathways are closely related to each other, with extensive mutual interference, and play a communication role in the immune-related microenvironment of tumors (**Figure 2**).

### Notch

Notch signaling regulates many aspects of cancer biology in the PDAC microenvironment and plays an important role in immunosuppression. It also takes a significant part in regulating the crosstalk between the different compartments of TME (147). The mechanism of Notch signaling involved in tumor vascular remodeling is that the overexpression of Jag1 in endothelial cells (ECs) leads to the increase of tumor vascular system, while the loss of Jag1 function in ECs leads to the decrease of vascular system and tumor growth. Dll4/Notch controls the emergence of endothelial cells in TME. Notch-mediated VEGFR2 inhibition maintains the stem cell phenotype and avoids overexpression, thereby controlling tumor vascular structure (148). By regulating the tip/stalk ratio, Notch is also implicated in regulating the escape of metastasizing cancer cells from dormancy, as tip cells are associated with this process. Notch signaling is involved in cell lineage regulation of lymphocyte development, regulation of B-lymphocyte subsets in the marginal region, and differentiation and function of DCs, innate lymphocytes, and helper and regulatory T cells (149). Notch signaling has been found to play an important role in the regulation of CD8+ cytotoxic T cell activation (150). This also suggests that Notch signaling plays a positive role in modulating the antitumor activity of CD8+T cells, but it is also critical in modulating different components of immunosuppression. For example, Notch is important for the differentiation of TAMs, mainly because TAMs show transcriptional profiles associated with the Notch pathway, and Notch signaling is involved in the increased phenotype of M1 macrophages (151). Deletion of CBF-1/Su(H)/LAG1 (CSL) transcription factor in mononuclear cell lines can block TAMs differences and TAM-related immunosuppression functions (152). As mentioned above, CAFs express activating markers such as α-SMA, fibroblast activating protein, and many secretory factors that are involved in cell recruitment during extracellular matrix remodeling and immunosuppression. Notch1 is a major regulator of senescence secretion in fibroblasts, and Notch signaling in fibroblasts has a synergistic effect on the inhibition of tumor formation, possibly because the loss of Notch canonical signal is associated with CAFs differentiation (153, 154).

### Fas/FasL

It was found that Fas/FasL pathway is mainly involved in mediating the apoptosis induced by cytotoxicity during T-cell development, while PDAC tumor cells antagonize the apoptosis of CD8+T cells in pancreatic cancer through Fas/FasL pathway, which leads to the down-regulation of Fas expression and the obstruction of the killing pathway of effector T cells to tumor cells, resulting in drug resistance (155). Kaplan-meier survival analysis showed that high levels of Fas cytoplasmic expression in PDAC cells were significantly associated with better prognosis (156).

### Hh

Hedgehog (Hh) signaling mediates PDAC immune-related behavior, mainly through the regulation of CD44+CD24+epithelial-specific antigen (ESA)+ pancreatic cancer stem cells (157). The main mechanism is that overexpressed sonic Hh (Shh) in CSCs affects the transcription factor NF-κB through autocrine and paracrine pathways and secretes tumor growth factors against PSCs in the tumor-associated stroma to control inflammatory responses and immunosuppression in TME (158, 159). It has been reported that inhibition of Hh signaling can inhibit the self-renewal of pancreatic CSCs and reverse chemotherapy resistance (160).

### TGF-β

Transforming growth factor-Beta (TGF-β) and it signaling pathway are key regulators of PDAC proliferation and differentiation, and their effects depend on TME. Changes in the microenvironment feedback to TGF-β signaling pathway may lead to tumor cells apoptosis or progression resulting in immunosuppression, they are generally viewed as separate fates for TGF-β-stimulated tumor cells, and opposite poles of the duality of TGF-β in cancer, or induce an EMT that promotes tumor invasion and metastasis and promote CSCs heterogeneity and drug resistance (161, 162). TGF-β is the major tumor suppressor signal of the pancreas, and its mechanism is that TGF-β activates Smad2 and Smad3 transcription factors (TFs) through membrane receptor kinases, which bind Smad4 to respond to the antitumor effects of immune cells (163). PDAC cells directly or indirectly induce EMT production by silencing TGF-β receptors or inactivation of Smad genes, promoting tumor aggressiveness and stem-like characteristics (164–166).

### Wnt

Abnormal activation of Wnt/β-catenin signal transduction leads to the accumulation of β-catenin in the nucleus and promotes the transcription of many oncogenes, including c-Myc and CyclinD-1, and is involved in the initiation, progression, dormancy, immunity, and stem cell maintenance of pancreatic malignancies (167). In canonical Wnt pathway, β-catenin, and T-cell factor (TCF)/lymphoid enhancement factor (LEF) has been identified as signal transducers of the canonical Wnt pathway, in which β-catenin is a core molecule (168–171). β-catenin promotes the progression of tumors *via* suppressing the T-cell responses (172). When referring to tumor growth, Wnt/β-catenin signaling may play opposing roles in different tumor tissues. It has been reported that inhibition of β-catenin signaling could suppress pancreatic tumor growth by disrupting the nuclear β-catenin/TCF1 complex (173). As the main cause of tumor recurrence, Wnt signaling is often involved in the activation of tumor dormancy. β-catenin expression up-regulates urokinase plasminogen activator (uPA) expression and promotes invasion, metastasis, and dormancy of tumor cells (174). In addition, Wnt/β -catenin signaling is also involved in the regulation of tumor immunity. Activation of β -catenin in tumors mainly excludes T cells from infiltrating the TME. Thus, activation of beta-catenin may represent a mechanism for primary resistance to T cell immune tumor therapy (175). Cross-priming contributes to the production of anti-tumor CD8+T cells. β -catenin expression in DCs negatively regulates anti-tumor immunity by inhibiting its cross-priming ability, thereby inhibiting CD8+T cell response. Therefore, the Wnt/β -catenin signaling pathway may be a potential target for anti-tumor immunotherapy (176).

### Hippo

Hippo signaling is a key regulator of organ size, tissue hemostasis and regeneration. Dysregulation of the Hippo pathway has been recognized in PDAC. YES-associated protein (YAP) and transcriptional coactivator with PDZ-binding motif (TAZ) are the two major downstream effectors of the Hippo pathway. YAP and TAZ regulate the behavior of pancreatic stellate cells and the recruitment of TAMs and MDSCs. Among them, YAP plays a dominant role, whose activation is associated with immune dysfunction and induces the recruitment of myelogenous suppressor cells, while counter-inhibition supports the infiltration of antigen-presenting macrophages and the activation of effector T cells, leading to immunosuppression and progression (177, 178).

## Clinical application and progress of immunotherapy

In today’s medical era, PDAC, as an incurable malignant tumor, escapes from chemotherapy or targeted therapy due to inherent genomic instability (179). Due to the instability of pancreatic cancer treatment, it is more likely to lead to resistance to surgical treatment, chemotherapy, and radiotherapy, at this time immunotherapy has become the fourth cornerstone of treatment. Immunotherapy is the best hope for preventing relapse and prolonging long-term survival by adapting the long-term memory function of the immune system (180, 181). Immunotherapy and its effect on treatment have been a hot topic in clinical research and may improve the prognosis of PDAC, mainly including targeted therapy and anti-tumor vaccination (182). It can also be divided into active or passive immunity according to the involvement of the host immune system (183). According to the different mechanisms of tumor activation, it can be divided into four categories: specific active immunotherapy, specific passive immunotherapy, non-specific adoptive immunotherapy and non-specific immunoregulation (184, 185). Immunotherapy for PDAC is a growing concern because conventional therapies such as chemotherapy are not effective in improving overall survival outcomes in patients with PDAC. However, it is still not a good solution for prolonging patient survival due to its unique tumor microenvironment and low tumor immunogenicity. Therefore, inducing more intratumor effector immune cells and reversing immunosuppression are the core of PDAC therapy (186). Immunotherapy has relatively mild side effects and has shifted the focus of treatment from the tumor itself to the immune system of the host. Immune cells in the TME have a key role in the development and progression. To overcome the deficiency of PDAC immune recognition, clinical researchers have explored several immunotherapy approaches in the form of vaccines to enhance antigen presentation and drive the expansion of tumor-specific T cell clones (187) to initiate new or enhance existing immune responses. Strategies for PDAC associated antigens (including telomerase (188), KRAS (189, 190), gastrin (191), CEA (192), MUC1 (193), and mesenchymal (194, 195)) include peptide-based vaccines (188–190), virus-based vaccines (196), listeria-based vaccines (195), DNA-based vaccines(neoantigens) (197, 198), and cell-based vaccines (199). Specific active immunotherapy mainly comprises therapeutic cancer vaccines, which aim to activate the immune system to eventually result in the expansion of tumor-specific T/B cells (200). Cancer vaccines can be generally divided into three major categories: cell-based vaccines, protein/peptide vaccines and genetic vaccines. Various genes or proteins have been targeted in recent I/II/III clinical trials of PDAC vaccines, such as telomerase and Wilms tumor gene (201, 202). Based on the results of many studies, vaccination methods have now been established to produce antigen-specific immune responses in PDAC patients (195, 203). Specific passive immunotherapy involves the direct infusion of tumor-specific immune effector cells or antibodies into a PDAC patient to mediate the immune response to the cancer. Monoclonal antibodies are currently the most widely adopted specific passive immunotherapy, including anti-EGFR and anti-VEGF antibodies, such as Erlotinib and Bevacizumab. A PD-1/PD-L1 blockade falls into this category of immunotherapy. A few studies have indicated that gemcitabine plus erlotinib shows favorable efficacy and safety and could significantly improve survival (204). Nonspecific adoptive immunotherapy involves the adoptive transfer of treated highly specific immune cells or cytokines into the patient to induce a passive immune response. In an early study, patients with advanced PDAC who received adoptive immunotherapy using intra-portal infusion of lymphoid-activated killer cells showed a lower rate of liver metastasis and a higher 3-year survival rate (205). Another study showed that adoptive immunotherapy of CTLs stimulated cells induced by co-culture with peripheral blood mononuclear cells and activated YPK-1 cells significantly inhibited postoperative liver recurrence of PDAC (206). Another immunotherapy direction for pancreatic tumors is to block the differentiation of macrophages induced by Galectin-9 (gal-9) (207), thereby enhancing the original immune response. Gal-9 is a member of the lectin β-galactoside-binding family and is highly expressed in PDAC. Binding of dectin-1 (208), an important natural immune receptor on the surface of macrophages, to Galectin-9 induces the transformation of macrophages to the M2 phenotype. Blocking galectin-9 can lead to reversal of immunosuppression, activation of CD4+ and CD8+ T cell effect and enhancement of anti-tumor effect, thus enhancing the killing effect of immune cells on PDAC cells and inhibiting growth and metastasis (207, 209). In addition, according to the immunosuppressive properties of NetG1+ CAF mentioned above, NetG1 is generally considered as a potential target for PDAC immunotherapy. In clinical application, inhibition of metabolic proteins in CAFs changes its immunosuppressive ability by blocking the NetG1 pathway with a neutralizing antibody (93). Therefore, immunotherapy is combined with other therapies such as surgery, chemotherapy, radiotherapy, targeted therapy, and other immunotherapies. Multiple therapies are used together to overcome resistance to immunotherapy (6, 210). Clinical trials have shown that a notable outcome in PDAC combination therapy is the combination of oxaliplatin (OXA) and gal-9 siRNA. OXA, part of folfirinox regimen for PDAC, triggers immunogenic cell death (ICD) effects in pancreatic tumor sites and kills tumor cells by inhibiting DNA synthesis and repair. OXA combined with gal-9 siRNA can block the galectin-9/Dectin-1 axis, reverse the immunosuppression induced by M2-TAMS, improve the efficiency of chemotherapy drug delivery and increased infiltration of antitumoral cytotoxic T lymphocytes, reversed immunosuppression, which has a significant effect on improving the quality of life of patients with advanced PDAC and controlling progression (207, 211). In terms of the current development of immunotherapy, immune checkpoint inhibition of T cells is a new technique in immunotherapy of PDAC, immune checkpoint inhibitors targeting cytotoxic T lymphocyte-associated protein 4 (CTLA-4) and programmed cell death protein-1 (PD-1)/programmed cell death ligand-1 (PD-L1) pathways have shown significant potential in the treatment (210, 212, 213).

### CTLA-4

CTLA-4 is an immune checkpoint receptor expressed on Tregs that activates conventional T cells (214). It’s also a negative regulator of T cell activation, also known as CD152. CTLA-4 is homologous to CD28 and has the same ligand. Both B7-1 (CD80) and B7-2 (CD86) ligands are expressed on antigen presenting cells (APCs) and can provide costimulatory signals to T cells. After these processes, the activated T cells expressed CTLA-4 on their surfaces. In addition, the binding of CTLA-4 to B7 inhibited T cell activation. CTLA-4 has a significantly higher affinity for B7 ligand than CD28. So, the CTLA-4 junction delivers inhibitory signals to T cells, while CD28 delivers stimulative signals (215, 216). In PDAC, anti-CTLA-4 antibody can block the interaction between CTLA-4 and B7, block the inhibitory signal, and down-regulate the immune system, to induce the host’s inhibitory effect on tumor and produce a lasting anti-tumor effect (217, 218). However, since T cell rejection is evident in PDAC, effector T cells are usually few in tumor tissue and confined to peripheral lymph nodes and lymphoid aggregates in PDAC (219). Therefore, the use of CTLA-4 antibody alone in the treatment of PDAC has little effect (220). This might be due to high tumor burden and the intrinsic nonimmunogenic nature of pancreatic cancer that cause immune quiescent, and the blockage of only one checkpoint is not enough for immunosuppressive reduction. And, with the development of clinical drug trials in recent years, the combination of CTLA-4 blocking drugs with surgical treatment or chemotherapy has gradually become a promising method in the treatment of PDAC.

### PD-1/PDL-1

In the immunotherapy of pancreatic tumors, the inhibitory effect of PD-1 and PD-L1 on tumors has always been the expected result of clinical studies. PD-1 plays an important role in inhibiting immune response and promoting self-tolerance by regulating T Cell activity, activating antigen-specific T Cell apoptosis, and inhibiting regulatory T Cell apoptosis. PD-L1 is a transmembrane protein, which is regarded as an inhibitory factor of immune response. It can bind to PD-1, reduce the proliferation of PD-1 positive cells, inhibit the secretion of cytokines, and induce cell apoptosis. PD-L1 also plays an important role in various malignant tumors by weakening the host’s immune response to tumor cells. Based on these ideas, the PD-1/PD-L1 axis is responsible for cancer immune escape and has a huge impact on cancer treatment (221). These molecules can be blocked by monoclonal antibodies, such as ipilimumab, nivolumab and pembrolizumab. However, immune checkpoint suppression monotherapy may be ineffective in pancreatic cancer, possibly due to low PD-L1 expression, highly complex interactions between tumor and stroma, and connective tissue hyperplasia. PD-L1 was confirmed to increase T-cell apoptosis *in vitro* and *in vivo* and to protect tumor cells from being killed, which unlocked the door of T-cell-based cancer immunotherapy (222). The PD-1/PD-L1 pathway has become popular in immunotherapy for the following reasons. Multiple studies have shown that PD-1 can inhibit the overactivation of immune responses and help maintain immune tolerance to autoantigens (223–225). After antigen recognition, PD-1 was expressed on the surface of activated T cells. However, PD-L1 is expressed by a variety of cell types, including immune and tumor cells, after interacting with cytokines such as IFN-γ, which is produced by activated T cells (224, 226). PD-1 and PD-L1 (B7-H1) belong to CD28+ superfamily and B7 superfamily respectively (227). Preclinical studies and clinical trials in mouse models have recently evaluated anti-PD-1/PD-L1 as an immune checkpoint blocking therapy for overcoming fatal malignancies. Anti-PD-1/PD-L1 antibodies have been shown to have clinical efficacy in many cancer types (228, 229). In 2007, PD-L1 was first considered as a new prognostic factor for patients with pancreatic cancer, when PD-L1 was first demonstrated to be up-regulated in PDAC specimens (230, 231). It has been suggested that PD-L1 blockers can effectively inhibit preestablished pancreatic cancer in mouse models by increasing IFN-γ production and decreasing IL-10 production (232). In addition, the level of tumor infiltrating Tregs in PD-L1 positive tumors was higher than that in negative tumors (227). These results provide a theoretical basis for the treatment of PDAC by targeting PD-1/PD-L1 pathway. But in clinical practice, the efficacy of PD-1/PD-L1 blockers alone may be limited for two main reasons. First, immunosuppression due to high tumor load is the reason why PD-1/PD-L1 blockade alone does not cure PDAC. Second, PDAC is nonimmunogenic in nature (233). Immune resistance is also responsible for the failure of anti-PD-1/PD-L1 monotherapy in pancreatic cancer. Like chemotherapy resistance, immune resistance to immunotherapy (including PD-1/PD-L1 blockade) can be divided into two types: primary resistance and acquired resistance. Primary drug resistance is a clinical condition in which the cancer does not respond to initial immunotherapy. Acquired drug resistance is a clinical condition in which some initial responders’ relapse after a period of response (179). Adaptive resistance is a mechanism of resistance in which a cancer cell is recognized by the immune system, but it protects itself against immune attack by the immune system. PDAC is resistant to therapy, including immune checkpoint inhibitors. In addition, pancreatic tumors appear to evade the immune response by inducing development of immunosuppressive T cells. Current studies indicate that both internal and external factors of tumor cells are involved in the regulatory mechanism of drug resistance. The failure of T cell recognition due to tumor antigen deficiency has become the most direct factor to predict the lack of response to anti-PD-1/PD-L1 therapy (234). In the immunotherapy of PDAC, PD-L1 can be used as a biomarker to predict the anti-PD-1/PD-L1 immunotherapy response rate. Since tumor-associated PD-L1 has been shown to increase T cell apoptosis *in vitro* and *in vivo*, and is expressed in most cell types after IFN-γ treatment, it can represent effector T cell activity (235). PDAC patients with high PD-L1 levels may not respond to PD-L1 therapy, due to the surge in the number of tumor immunosuppressive cells, such as the high infiltration of Tregs/MDSCs/TAMs, where TGFβ stimulates dendritic cells to induce immunosuppression (236). And CSF-1R activates TAMs as a macrophage colony-stimulating growth factor receptor (179). Therefore, PD-L1 should not be the only predictive biomarker. Other biomarkers that affect TME and reflect effector T cell function, Tregs, MDSCs, and TAMs should be combined with PD-L1 to improve the accuracy of predicting anti-PD-1/PD-L1 immunotherapy responses. Therefore, accurate identification of tumor immune characteristics will help predict the response to anti-PD-1/PD-L1 therapy (210). The current studies indicate that a PD-1/PD-L1 blockade in combination with surgery, chemotherapy, radiotherapy, molecular targeted therapy, or other immunotherapies could modulate the immunoediting process, TME and the immune response in pancreatic cancer, which have been or will be validated in the latest clinical trials.

### Drug combinations in immunotherapy

Drug combination in immunotherapy has always been a hot project in clinical practice (**Table 1**). Multiple drugs combined with each other can more effectively prevent PDAC from progressing due to treatment resistance. Among the drug combinations for PDAC, the most common is the combination of anti-PD-1/PD-L1 therapy with chemotherapy. Gemcitabine and 5-FU remain the main adjunctive treatment strategies for PDAC in clinical practice (237). Chemotherapeutic drugs can inhibit tumor growth by encouraging tumor cells to release antigens and reactivate anti-tumor immune responses (238). Immunotherapeutic agents are added to chemotherapy to enhance and synergistically enhance the anti-tumor effect of the two therapies (229). In addition, current studies have shown that specific molecules targeted against PD-1/PD-L1 therapy can simultaneously improve chemotherapy sensitivity and inhibit immunosuppression. Relevant data also clearly demonstrated that in the treatment environment of PDAC, anti-PD-1/PD-L1 therapy combined with chemotherapy (related clinical studies of various types of drugs) effectively extended the later survival rate of patients. In mouse models, the role of programmed cell death neutralizing antibody 1 (PD-1) and an OX40 agonist (which provides survival signals to activated T cells) in PDAC was evaluated. The combination of anti-PD-1 inhibitory and anti-OX40 agonist antibodies reduces the proportion of T-regulatory and exhausted T cells in PDAC and increases numbers of memory CD4+ and CD8+ T cells, eradicating all detectable tumor. This information can be used in development of immune-based combination therapies for PDAC (239). With the diversification of treatment methods, the combination of immunotherapy and targeted therapy has shown good results. Although targeting growth factor receptor inhibitors such as anti-EGFR antibodies (erlotinib) has significantly improved survival in pancreatic cancer, only a small population have benefitted (240). Various preclinical studies and clinical trials have shown that the frequency of BRCA1/2 mutations in pancreatic cancer is between 4% and 7% (241, 242). Poly (ADP-ribose) polymerase (PARP) inhibitors are a class of small-molecule drugs that inhibit the activity of PARP and may lead to cell death lacking homologous recombination repair (243). It is common in PDAC cells with BRCA1/2 mutations. A recent study confirmed the efficacy and safety of PARP inhibitors in PDAC (244). Therefore, anti-PD-1/PD-L1 combined with PARP inhibitors may be effective against PDAC with BRCA1/2 mutations. And it has been suggested that if agents could be developed to inactivate the WNT-β-catenin signaling pathway and prevent T-cell infiltration, it would be effective if combined with a PD-1/PD-L1 blockade (175). In immunotherapy, a specific method has been gradually used in clinical treatment in recent years. Based on the different mechanisms of various immunotherapies, anti-PD-1/PD-L1 therapy combined with other immunotherapies can often regulate TME and immune response and improve the anti-cancer efficacy. For example, combining cancer vaccines with other immune checkpoint inhibitors. Multiple clinical trials have shown that the combination of anti-PD-1/PD-L1 therapy with other types of immunotherapy is effective in treating PDAC, and the combination of GVAX (GM-CSF cell-based vaccines) vaccine and PD-1 blocker can significantly improve the survival of patients compared with anti-PD-1 or GVAX monotherapy (245). This is because GVAX in combination with PD-1 blockers increases CD8+TILs in PDAC and promotes the activation of CD8+T cells, resulting in the production of more tumor-specific IFN-γ in TME, which will help overcome Tregs, CTLA-4, and PD-1/PD-L1 immunosuppressive pathways (246).

**Table 1:** Immune checkpoint inhibitors in combination with other therapies currently used in the clinical test medication.

Target	Trade name	Stage	Company	Combination therapy	Current phase	Adverse effects
PD-1	Pembrolizumab (MK-3,475)	FDA-approved	Merck	capecitabine	I	alopecia, diarrhea, vomiting, abdominal pain , marrow-suppression, acro-anesthesia, erythra, oral ulcer
				gemcitabine, nab-paclitaxe	Ib/II	
				gemcitabine, reolysin, 5-FU, irinotecan	II	
				acalabrutinib	II	
	Nivolumab (BMS-936,558;MDX1106;ONO-4,538)	FDA-approved	Bristol Myers Squibb	gemcitabine, nab-paclitaxel	I	marrow-suppression, alopecia, abdominal pain, anemia, nausea, constipation, renal failure
				gemcitabine, nab-paclitaxel, cisplatin, paricalcitol	I/II	
				cabiralizumab, gemcitabine, nab-paclitaxel	I/II	
CTLA-4	Tremelimumab	Phase I–III	AstraZeneca	gemcitabine	Ib	pruritus, alopecia, arrhythmia, pyrexia, marrow-suppression
	Ipilimumab	FDA-approved	Bristol Myers Squibb	gemcitabine	Ib	
PD-L1	Durvalumab	Phase I	AstraZeneca	epacadostat	I/II	feeble, pneumonia, mucosal edema, cephalalgia, alopecia
	Atezolizumab (MPDL3280A)	Phase I–III	Roche	selicrelumab, gemcitabine, nab-paclitaxel	Ib/II	
	Avelumab (MSB0010718C)	Phase I–III	Pfizer	gemcitabine, nab-paclitaxel, hydroxychloroquin	II	

1. reolysin: a reovirus with potential oncolytic activity.

2. selicrelumab: CD40 agonist.

3. cabiralizumab: anti-CSF-1 receptor.

4. paricalcitol: D-vitamin analog.

5. acalabrutinib: Bruton tyrosine kinase inhibitors.

In recent decades, encouraging results have been achieved in mouse models and clinical trials, and in various preclinical studies on the efficacy of immunotherapy and its potential therapeutic application in pancreatic cancer. The understanding of the biology and pathophysiology of TME in PDAC has improved significantly through in-depth research, and new immunotherapy approaches are emerging that may help improve the devastating prognosis of patients with PDAC.

## Conclusions and perspectives

In PDAC, immune-related cells and cytokines in tumor microenvironment show tumor-promoting and tumor-suppressive activity to varying degrees. Tumor-promoting cells include MDSCs, CAFs, TAMs, etc., while tumor-suppressive immune cells include cytotoxic T cells (CTLs) and natural killer cells (NK cells). These cells recognize and to varying degrees alter tumor cells and their cellular environment with cytokines produced by paracrine and autocrine, which is also named tumor immune microenvironment (TIME). It is particularly important to accurately understand the immune components-related signal transduction pathways, in which the role of the signaling pathways involved in different parts of the TME is a prerequisite for better understanding of preclinical and clinical model data to design better treatments for signaling pathway targets in PDAC. In the treatment of PDAC, traditional chemotherapy has been regarded as an effective method of disease remission, but clinical data show that the effectiveness is only limited to a subset of patients. Therefore, immunotherapy has become a popular direction of clinical research, with its advantages of persistence, universal applicability, and relatively low toxicity. Checkpoint inhibitors have always been a hot spot in clinical research and application. In addition, the following clinical experiments show that PDAC immune checkpoint inhibitors combined with chemotherapy or radiotherapy can enhance the sensitivity of radiochemotherapy, reduce the toxic and side effects of radiochemotherapy, improve the efficacy of radiochemotherapy and reduce the occurrence of treatment resistance. Although immunotherapy is not an all-powerful treatment, it opens a new field in the treatment of PDAC with a very high degree of malignancy. With the progress of drug clinical research, immunotherapy will become one of the main treatment methods for tumors. Given the advances in our understanding of the PDAC microenvironment and emerging strategies, we have reason to be hopeful for the successful treatment of PDAC in the future.

## Author contributions

JZ completed the main content of the article. SH and RL gave guidance and corrected the mistakes. All authors contributed to the article and approved the submitted version.

## Conflict of interest

The authors declare that the research was conducted in the absence of any commercial or financial relationships that could be construed as a potential conflict of interest.

## Publisher’s note

All claims expressed in this article are solely those of the authors and do not necessarily represent those of their affiliated organizations, or those of the publisher, the editors and the reviewers. Any product that may be evaluated in this article, or claim that may be made by its manufacturer, is not guaranteed or endorsed by the publisher.
